# Regulation of straw decomposition and its effect on soil function by the amount of returned straw in a cool zone rice crop system

**DOI:** 10.1038/s41598-023-42650-9

**Published:** 2023-09-21

**Authors:** Lin Liu, Ming Cheng, Lei Yang, Xinyue Gu, Jingyi Jin, Minjie Fu

**Affiliations:** 1 School of Agriculture, Yanbian University, Yanji, 133002 China; 2 College of Resources and Environment, China Agricultural University, Beijing, 100193 China; 3 Research Center of Chemical Biology, Yanbian University, Yanji, 133002 China

**Keywords:** Ecology, Microbiology, Biogeochemistry, Ecology

## Abstract

The degradation process of returned straw in rice fields can improve soil organic matter and promote sustainable agriculture. The degradation process of returned straw is a humification process as well as a mineralization process involving microorganisms and enzymes. However, the degradation process of returned straw, the effect on straw decomposing microorganisms and the regulatory mechanism on potential functionality under cool climate flooding conditions are currently unknown.For this purpose, we investigated the biodegradation of straw from a biodegradation point of view at 20, 40, 71, 104, and 137 d after return under conventional (130 kg hm^−2^), 1/3 straw return (2933 kg hm^−2^), 2/3 straw return (5866 kg hm^−2^), and full straw return (8800 kg hm^−2^) applications in cool climate rice fields.. The test found *Paludibacteraceae* and *Archaeaceae* were the dominant bacteria for straw degradation, and their relative abundance was highest when 2/3 of straw was returned to the field. The straw degradation extracellular enzyme activity was higher in the late return period (104 d). At this time, the potential functionality of the soil differed significantly among the different return amounts, with the best extracellular enzyme activity and potential functionality at the 2/3 straw return amount. Therefore, the optimal amount of rice straw returned to the field is 5866 kg hm^−2^ at the current conventional N application rate (130 kg hm^−2^) in the cold zone.

## Introduction

Straw is an important by-product of agricultural production, accounting for about 50% of the total crop^1^. China is a large agricultural producer and ranks first in the world in terms of the amount of straw each year^2,3^. As an important renewable resource, straw contains mineral nutrients and large amounts of organic matter required for plant growth, and is an important source of organic fertilizer for soil fertilization^4^. Therefore, straw return to the field is of great significance to promote sustainable agricultural growth and green agriculture in China. Nutrient release from returned straw is a complex and slow process, and in view of this, it is necessary to elucidate the dynamics of straw mass and the number of actors (e.g., enzymes, bacteria) in the decomposition of straw from the perspective of biodegradation^5^.

The degradation process of returned straw is a mineralization and putrefaction process involving both microorganisms and enzymes^6^ and is divided into four main stages: decomposition of the carbon fraction in an easily mineralized state, decomposition of hemicellulose, decomposition of cellulose, and decomposition of lignin^7,8^. The degradation microorganisms during straw degradation vary according to the straw residue, in which bacteria are active in the straw degradation process^9–11^. Extracellular enzymes are key factors for all biochemical processes in soil and are closely related to environmental quality and nutrient environment, and the dynamics of these indicators reflect the decomposition capacity during straw degradation^12^. At the same time, the extracellular enzyme activity and bacteria during straw degradation are highly susceptible to external mitigating factors such as soil type, temperature and water content due to the planting area, planting method, aerobic and anaerobic environmental conditions in rice cultivation^13^.

It has been suggested that the amount of straw returned to the field is an important field management factor affecting the rate of straw degradation^14,15^. An appropriate amount of straw return can lead to improvement in the potential function of the soil. Too much straw returned to the field can easily lead to insufficient nitrogen fertilizer application and increased pests and diseases^16^. Therefore, it is not better to return more straw to the field, but the amount of straw to the field needs to be determined by the condition of the soil itself and climatic factors. In addition, straw degradation is inhibited because clay and heavy soil particles under flooded conditions provide better physical protection for plant residues directly^14,17^. Prolonged anaerobic soil environment in rice fields can affect microbial growth and inhibit straw degradation, which can easily lead to incomplete decomposition^18,19^. Currently there is a lack of systematic knowledge about the biological characteristics of straw degradation in returned fields under flooded conditions in the cooler regions of northeast China. Therefore, it is necessary to further investigate the changes in enzyme activity, bacterial community structure and metabolic capacity associated with straw degradation in cold and cool zone rice fields at different stages of decomposition.

Therefore, this experiment was conducted under annual rice cultivation conditions in the cool zone of northeast China using nylon mesh bag in situ culture, with the aim of exploring the effects of different straw returns on (1) straw decomposition rate, material composition and representative enzyme activities associated with straw decomposition; (2) straw-related bacterial community structure and metabolic activity; and (3) soil multifunctionality from the perspective of biodegradation effects (3) impact on soil multifunctionality from the perspective of biodegradation effects. In order to provide a reference basis for constructing the degradation mechanism of straw returned to the field in the cool zone.

## Materials and methods

### Description of the test site

The study site was located at the experimental base of Yanbian University in Longjing City, Jilin Province (42°46′18″N, 129°23′46″E), where a single-season rice-winter recreational cropping system was used. The test area was located in the mid-temperate monsoon climate zone, with an average annual precipitation of 549.3 mm, an average annual temperature of 5.6 ℃, a frost-free period of approximately 126 d, and an annual active temperature of approximately 2650 ℃, which belongs to the cool northeast region. The test rice soil was black soil, which was classified as soft soil (Mollisols) according to the USDA soil classification. The basic physical and chemical properties of the soil at the beginning of the experiment are listed in Table 1.

**Table 1:** Basic physicochemical properties of the test soil.

Soil horizon (cm)	pH	EC	Available K	NO_3_^-^-N	NH_4_^+^-N	Available P	Total K	Total N	Total P	Organic matter
		(μs cm^−1^)	(mg kg^−1^)	(mg kg^−1^)	(mg kg^−1^)	(mg kg^−1^)	(g kg^−1^)	(g kg^−1^)	(g kg^−1^)	(g kg^−1^)
0–15	6.12	131.03	485.00	23.46	69.35	33.17	16.71	0.73	1.12	62.91
15–30	6.69	82.27	227.00	22.35	40.22	19.19	19.21	0.49	0.95	59.53

### Experimental design

The straw decomposition test was carried out by nylon mesh bag layered in situ incubation method, and rice straw was cut into 5 cm lengths to simulate the state of straw after fragmentation, dried at 60 °C for 24 h, and then prepared for use. The amount of straw per unit area and tillage layer was calculated based on the local rice yield, grain to straw ratio, and the depth of 1/3, 2/3, and full amount of straw returned to the field. 4.58 g/bag (T1), 9.17 g/bag (T2), and 13.75 g/bag (T3) were weighed in nylon mesh bags (10 cm wide, 30 cm long, and 0.1 mm aperture) to simulate the actual agricultural time for 1/3, 2/3, and full amount of straw returned to the field, respectively. straw to the field. 1/3 straw to the field (2933 kg hm^−2^), 2/3 straw to the field (5866 kg hm^−2^), and the full amount of straw to the field (8800 kg hm^−2^), respectively.The nylon mesh bale allows free access to soil fines and microorganisms. The straw bales were buried diagonally at ∠45° in 0–15 cm of the soil before spring tillage (May 21, 2021) and ensured that the straw was evenly distributed in the bales to maximize contact with the soil. The test fertilizers were urea (46% N), diammonium phosphate (18% N, 46% P_2_O_5_), and potassium sulfate (50% K_2_O). Nitrogen (130 kg hm^−2^) was applied as 4:4:2 as base fertilizer: tiller fertilizer: spike fertilizer; phosphorus (P_2_O5, 70 kg hm^−2^) was applied as base fertilizer; potassium (K_2_O, 80 kg hm^−2^) was applied as 5:4:1 as base fertilizer: tiller fertilizer: spike fertilizer. Other field management measures were the same as local conventional management. Four straw bales were removed from each treatment replicate at 20 d (June), 40 d (July), 71 d (August), 104 d (September), and 137 d (October) after straw bedding, and the straw bales were transported to the laboratory via ice. A total of 60 straw bales were removed for measurement during the test period. The straw samples were stored at 4 and − 70 °C, respectively. The straw samples stored at 4 °C were used for relevant enzyme activity assays (within 24 h), and the samples stored at − 70 °C were analyzed for bacterial bioassays.

### Determination of straw composition, decomposition extracellular enzyme activity, and bacterial diversity

The content of soluble matter (Monosaccharides, disaccharides, and polysaccharides insoluble in water except for starch, cellulose, chitin, and hemicellulose.), cellulose, hemicellulose, and lignin in returned straw was determined by acid and alkaline liquid washing methods^20^.

The degradation rate of returned straw was determined by weighing method.The calculation formula is as follows:

$${\text{Cumulative}}\;{\text{degradation}}\;{\text{rate}}\;{\text{of}}\;{\text{straw (\% )}} = \frac{{{\text{Initial}}\;{\text{straw}}\;{\text{weight}} - {\text{Residual}}\;{\text{straw}}\;{\text{weight}}}}{{{\text{Initial}}\;{\text{straw}}\;{\text{weight}}}} \times 100{\text{\% }}$$

In this study, the activities of eight extracellular enzymes associated with C and N cycling and redox reactions of straw were determined (Table 2). Among them, hydrolytic enzymes associated with straw decay (β-glucosidase, α-glucosidase, β-xylosidase, β-cellulodiglucosidase, acetylaminoglucosidase, and leucine aminopeptidase) were determined using a fluorescent microplate enzyme assay technique^21–23^. Method: A fresh sample of straw equivalent to 0.2 g dry weight was weighed in 50 ml CH_3_COONa buffer solution (50 mM) and mixed thoroughly using a vortexer to maintain a homogeneous suspension. 10 mM control, 200 mM substrate, buffer, and sample suspension were quantitatively added into 96 ELISA plates in sequence and incubated at 25°C for 4 h with protection from heat. The signal was recovered at 450 nm using a fluorescence digitizer with excitation at 365 nm for analysis.

**Table 2:** Types of Extracellular Enzymes Related to Straw Decomposition and Their Related Substrates, Enzyme Function and Enzymes International System Classification Number (E.C).

Cor. cycle	Enzyme	Abbreviation	E.C	Substrate	Enzyme function
C cycling	α-glucosidase	αG	3.2.1.20	4-MUB-α-D-glucoside	Sugar degradation
	β-glucosidase	βG	3.2.1.21	4-MUB-β-D-glucoside	Sugar degradation
	β-cellobiosidase	CBH	3.2.1.91	4-MUB-β-D-cellobioside	Cellulose degradation
	β-xylosidase	βX	3.2.1.37	4-MUB-β-D-xyloside	Hemicellulose degradation
N cycling	N-Acetyl-glucosaminidase	NAG	3.2.1.30	4-MUB-N-acetyl-β-D-glucosanimide	Chitin degradation
	Leucine aminopeptidase	LAP	3.4.11.1	L-Leucine-7-amino-4-methyl coumarin	Protein degradation
Redox reaction	Phenol oxidase	Phox	1.10.3.2	L-DOPA	Lignin oxidation, toxicity reduction
	Peroxidase	Perox	1.11.1.7	L-DOPA	Lignin degradation

Cor. Cycle: an enzyme involved in this cyclic process; 4-MUB:4-methylumbelliferone; L-DOPA: L-3,4-dihydroxyphenylalanine.

Phenol oxidase and peroxidase assays were performed by spectrophotometric methods^24^.Method: Fresh samples of straw equivalent to 0.2 g dry weight was weighed in 50 ml CH_3_COONa buffer solution (50 mM) and mixed thoroughly with a vortexer to maintain a homogeneous suspension. 25 mM L-DOPA, 0.3% H_2_O_2_, buffer solution, and sample suspension were added to the enzyme standard plate in turn, and incubated at 25 °C for 20 h under protection from light and then measured at 450 nm using an enzyme standard. The determination was performed at 450 nm using an enzyme marker.

The straw decay-associated microorganisms were sequenced by 16SrDNA high-throughput sequencing: The samples were melted on ice, centrifuged, and mixed well. DNA was extracted from the straw microbiome using the PowerSoil DNA Isolation Kit (MoBio Laboratories, Inc., CA), and DNA quality and concentration were determined using a Nanodrop 2000 (ThermoFisher Scientific, Inc., USA). DNA quality and concentration tests were performed using Nanodrop 2000 (ThermoFisher Scientific, Inc., USA). The quality-checked samples were stored at − 20°C for subsequent experiments.

The straw sample DNA was used as a template and the region of determination was the bacterial 16S rDNA V3-V4 region. Primers 338F (5′-ACTCCTACGGGAGGCAGCAG-3′) and 806R (5′-GGACTACHVGGGTWTCTAAT-3′) were used for amplification. the PCR amplification system (25 μL) was: 12.5 μL 2xTaq Plus Master Mix, 1 μL each of upper and lower primers, 7.5-X μL double-distilled water, 3 μL of BSA (2 ngμL^−1^), X (30 ng) DNA template. The amplification procedure was: 94 ℃ for 5 min, 30 cycles 94 ℃ for 30 s, 50 ℃ for 30 s, 72 ℃ for 60 s, 72 ℃ extensions for 7 min, and end at 4 ℃. The PCR products were amplified on an ABI 9700 PCR instrument (ThermoFisher Scientific, Inc., USA), and the amplified target bands were detected by 1% agarose gel electrophoresis and purified using the AgencourtAMPure XP (Beckman Coulter, Inc., USA) nucleic acid purification kit. Purification.

The PCR products were used to construct microbial diversity sequencing libraries using the NEB Next Ultra II DNA Library Prep Kit (New England Biolabs, Inc., USA) for library construction, and the IlluminaMiseq PE300 (Ltd. performed Paired-end sequencing using IlluminaMiseq PE300 (Illumina, Inc., USA) high-throughput sequencing platform. The sequencing results were stored in the NCBI database (Accession number: PRJNA925293).

### Data processing and statistical analysis

Data were processed with Excel 2020 and line graphs were produced with Origin2021 to compare straw degradation rates, microbial α-diversity, and soil multifunctionality levels at different periods for different straw return treatments using the Duncan comparison method. Soil functionality was calculated using the averaging method (Z-score conversion)^25–27^, as this method can only provide interpretable results when the multifunctionality index is at a high level^28^, so the averaging method was used to analyze only the periods of high functionality (data at 104 d of straw return were chosen for this paper). The data were leveled using Qiime version v.1.8.0, and the least amount of sample data in this data was used for the calculation, and in this paper, OTUs were clustered using a similarity of 0.97 and subjected to Alpha diversity analysis^29^. Using statistical analysis, Barplot plots of species composition were drawn to observe the community structure and its variation at the sample family level^30^. PLS-DA was used to model the relationship between microbial content and sample class, and Beta diversity analysis was performed between groups. A partial least squares pathway model (PLS-PM) was constructed using the R language "Plspm" package to determine the relationship between microbial diversity and soil multifunctionality (based on averaging), and direct and indirect associations between bacterial abundance, bacterial composition, C-cycle related enzyme activity, N-cycle related enzyme activity, oxidation related enzyme activity, and soil multifunctionality were assessed.

### Ethical approval

All rice straw samples collected in this study have been licensed. All the rice straw experiments were in compliance with relevant institutional, national, and international guidelines and legislation.

## Results

### Changes in ambient temperature and soil water content during the growing season

**Figure 1:**
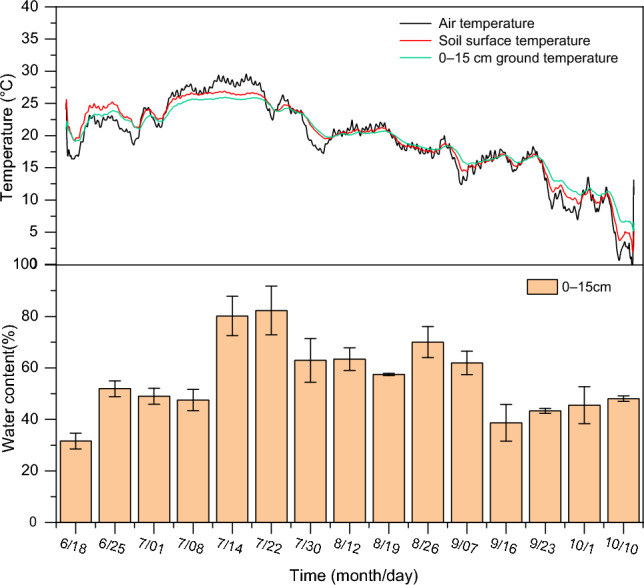
Variation of water content, temperature, and air temperature in each soil layer.

The air temperature and soil temperature of each layer varied greatly during the whole experiment period, and the air and soil temperatures were high in July, with the maximum air temperature reaching 30 °C and the average soil temperature of each layer reaching 25 °C. The difference between air and soil temperatures in each layer during this period was more pronounced, with the difference between the maximum air and soil temperatures reaching 10 °C in 0–15 cm (Fig. 1). In September, the temperature decreased significantly and was lower than the soil temperature, while the difference between the surface temperature and the 0–15 cm soil temperature became larger with the increase of the return time in this period. The soil moisture content of 0–15 cm fluctuated widely during the experiment, with the lowest soil moisture content of about 30% at the beginning of the return period (6/18) and the highest soil moisture content of about 80% on average in mid-to-late July, followed by a decreasing trend. The soil moisture content of 0–15 cm averaged 50% during the test period. The trends of soil temperature and soil water content were similar.

### Changes in degradation rate and composition of returned straw at different periods

The accumulated degradation rate of returned straw averaged about 48.86% during the test period, as shown in Table 3 The degradation rate was faster at the early stage of straw return (0–20 d), when the average degradation rate of straw was about 26.12%. At the later stage (104–137 d), the degradation rate was slow. The cumulative degradation rate of T3 was significantly lower than that of T1 and T2 at 40 and 104 d. During the test period, the cumulative degradation rates of all treatments were T2 > T1 > T3 at the same return time.

**Table 3:** Cumulative degradation rate of straw returned to the field in each period under different return quantities.

Sample	Cumulative degradation rate					Fitted the degradation equation
	20 d	40 d	71 d	104 d	137 d	
T1	28.13 ± 3.27a	37.13 ± 1.21a	39.01 ± 4.74a	46.54 ± 0.75a	49.32 ± 6.67a	y = ln(x − 4.67)
T2	29.45 ± 2.62a	36.52 ± 1.38a	42.58 ± 4.72a	48.88 ± 2.04a	54.37 ± 0.38a	y = ln(x − 7.23)
T3	20.78 ± 4.82a	29.21 ± 1.86b	35.67 ± 4.93a	40.00 ± 1.36b	42.90 ± 0.07a	y = ln(x − 7.59)

Lowercase letters indicate significant differences between treatments in the same period (*p* < 0.05).

**Figure 2:**
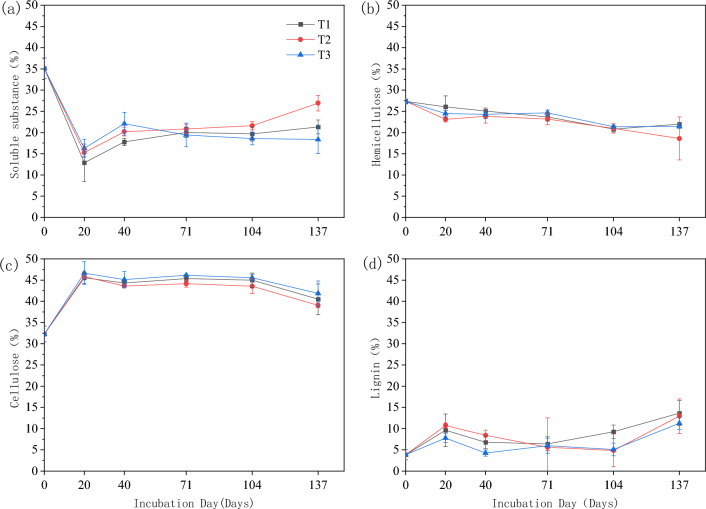
Composition of straw at different return times.

The highest content of cellulose was found in the straw at different periods, accounting for about 40–45% of the total straw, and the lowest content of lignin, accounting for about 5–15% of the straw (Fig. 2). In this experiment, the overall trend of hemicellulose and cellulose content in the straw residue was decreased. The content of hemicellulose in the residue of each treatment was similar in the middle of the return period (40–104 d), and the hemicellulose content of T2 was significantly lower than that of T1 and T3 in the late return period (137 d). the content of cellulose in each treatment decreased significantly in the early return period (20–40 d) and the late return period (104–137 d), and the difference in cellulose content in the straw residue during the test period was more obvious, with the treatments Soluble matter and lignin content showed an overall increasing trend, with a significant increase in soluble matter content in the early stage (20–40 d) and late stage (104–137 d). The soluble matter content of T3 was significantly higher than that of T2 and T3 before 71 d of field return, and the highest soluble matter content was found in T2 after 71 d of field return, and the differences were more obvious among treatments. The lignin content in the straw residue showed a trend of decreasing and then increasing, and the decrease of lignin content in the first stage of degradation (20–40 d) was about 5%, and the difference between treatments was obvious at this time, T2 > T1 > T3, and the increase of lignin content in the later stage of field repatriation (104–137 d) was about 10%, and the lignin content of T1 was significantly higher than the other two treatments, and reached the maximum at 137d of field repatriation.

### Changes in bacterial diversity of returned straw

**Figure3:**
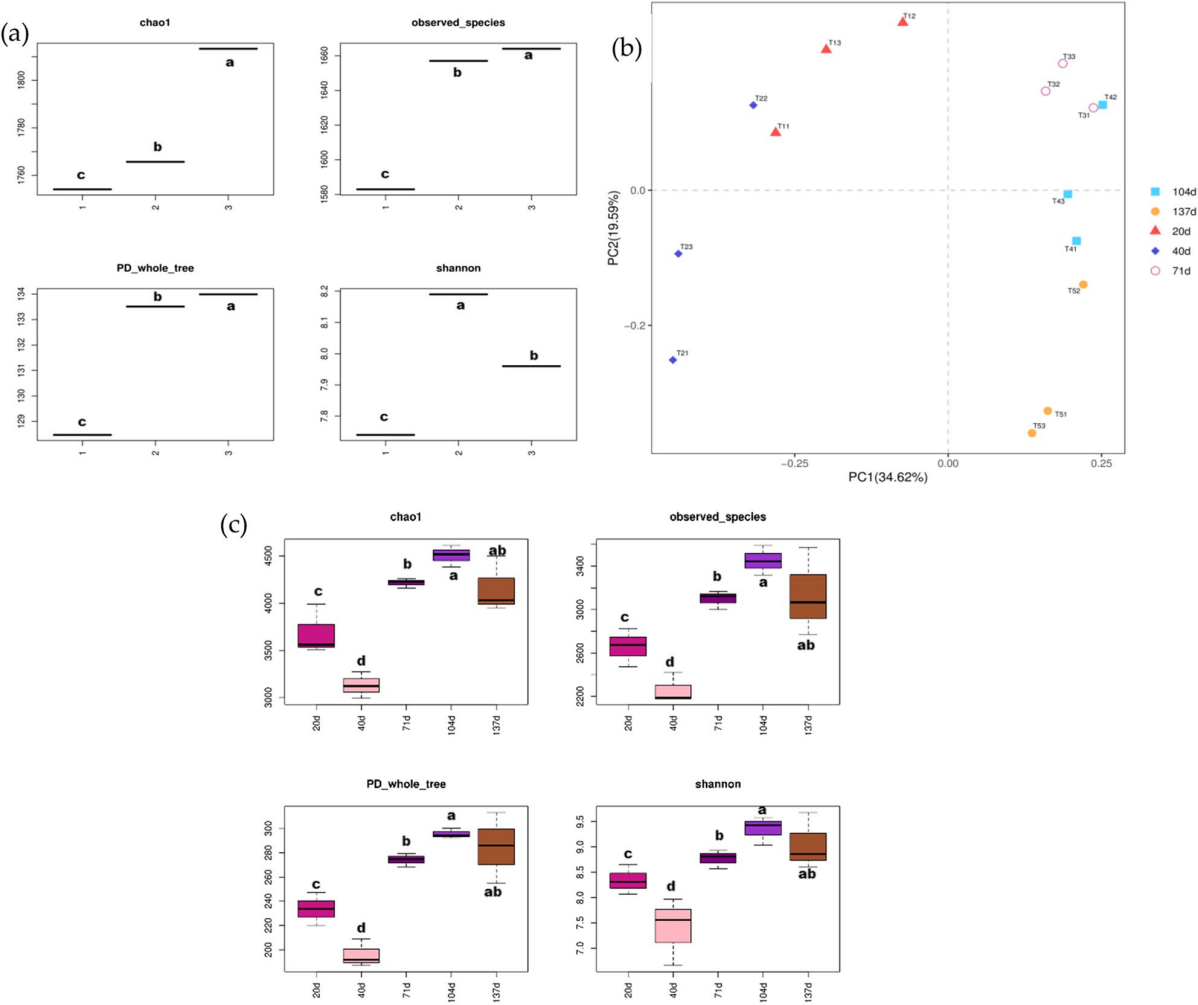
Bacterial diversity of returned straw. (**a**) shows the alpha diversity of different straw return amounts, and 1, 2 and 3 in the figure indicate T1, T2, and T3, respectively. (**b**) shows the PCA analysis of different treatments and different return times, and (**c**) shows the alpha diversity of bacteria at different return times of straw decomposition. The decimal digits in the treatment names indicate the decomposition time, i.e. 1, 2, 3, 4 and 5 indicate 20, 40, 71, 104, and 137 d, respectively, and the digits in the individual digits indicate the amount of straw returned to the field, i.e. 1, 2 and 3 indicate 1/3, 2/3 and the full amount of straw returned to the field, respectively. Lowercase letters in (**a**) (**b**) indicate significant differences (*p* < 0.05) among treatments with different straw return volumes and return times, respectively.

Chao1, observed species, PD whole tree, and Shannon were commonly used to measure microbial α-diversity. The values of chao1, observed species, PD whole tree often showed an increasing trend as the amount of straw returned to the field increased, reaching a maximum at T3, and the observed species, PD whole tree was similar at T2 and T3 (Fig. 3a). As the amount of straw returned to the field increased Shannon’s number showed a trend of increasing and then decreasing, specifically T2 > T3 > T1. T1 treatment had the lowest bacterial α diversity. During the decomposition of returned straw, the α diversity of microorganisms fluctuated greatly in each period, and the change trends of chao1, observed species, PD whole tree, and Shannon were consistent. α diversity of bacteria was higher in the early period (20 d), and gradually decreased with the increase of returning time (20–40 d), reaching the lowest value at 40 d, and decomposition The α diversity increased significantly in the middle stage (40–104 d) and reached the maximum value at 104d, and slightly decreased in the late stage (137 d) (Fig. 3c). The β-diversity of straw-degrading bacteria responded more strongly to the time of straw return compared to the amount of straw returned.The β-diversity of bacteria in the decomposition process of returned straw had a strong clustering mainly with the return time, with two groups of 20 and 40 d closer together, and three groups of 70 d, 104 d, and 137 d closer together. Within the same straw degradation time, the treatment with 2/3 of the straw returned to the field was more distant from the other two treatments (Fig. 3b).

### Changes in bacterial community composition of returned straw

**Figure 4:**
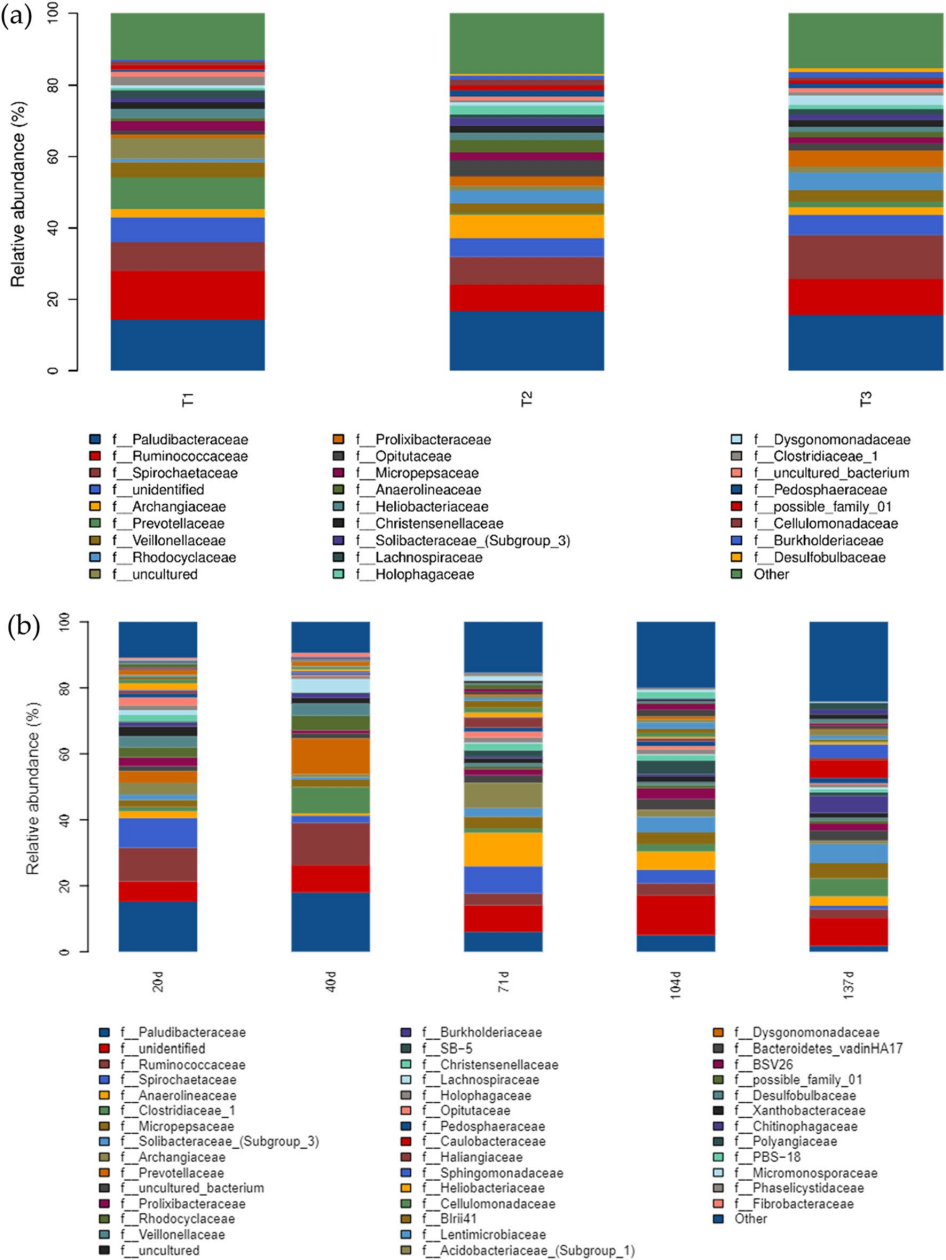
Bacterial species composition of returned straw. (**a**) indicates different treatments of straw-degrading bacteria for returning to the field; (**b**) indicates different straw-degrading bacteria for returning to the field at different times.

As shown in Fig. 4, the families *Palearobacteriaceae*, *Ruminobacteriaceae* and *Spirochaetes* were the dominant bacteria for degradation at the family level during the decomposition of the different treatments.The relative abundance of *Paludibacteraceae* and *Archangiaceae* at T2 was the highest. The relative abundance of *Paludibacteraceae*, and *Archangiaceae* was the highest, whereas the relative abundance of *Archangiaceae* was about three times higher than that of the T1 and T3 treatments. The relative abundance of *Ruminococcaceae* was the highest in the T1 treatment, which was about two times higher than that of the T2 treatment. The relative abundance of *Spirochaetaceae* was the highest in the T3 treatment (Fig. 4a). The relative abundance of *Paludibacteraceae* was the highest at 40 d and showed an overall decreasing trend with increasing decomposition time in the middle and late stages (71–137 d) of straw decomposition. The relative abundance of *Ruminococcaceae* was higher in the early stage (20–40 d) (Fig. 4b). The relative abundance of *Spirochaetaceae* was higher at 20 and 71 d and lowest at 137 d. The relative abundance of *Anaerolineaceae* was higher in the middle and late stages of field return (71–137 d), with the highest relative abundance of *Anaerolineaceae* at 71 d of field return.

### Changes in extracellular enzyme activity of returned straw in different periods

**Figure 5:**
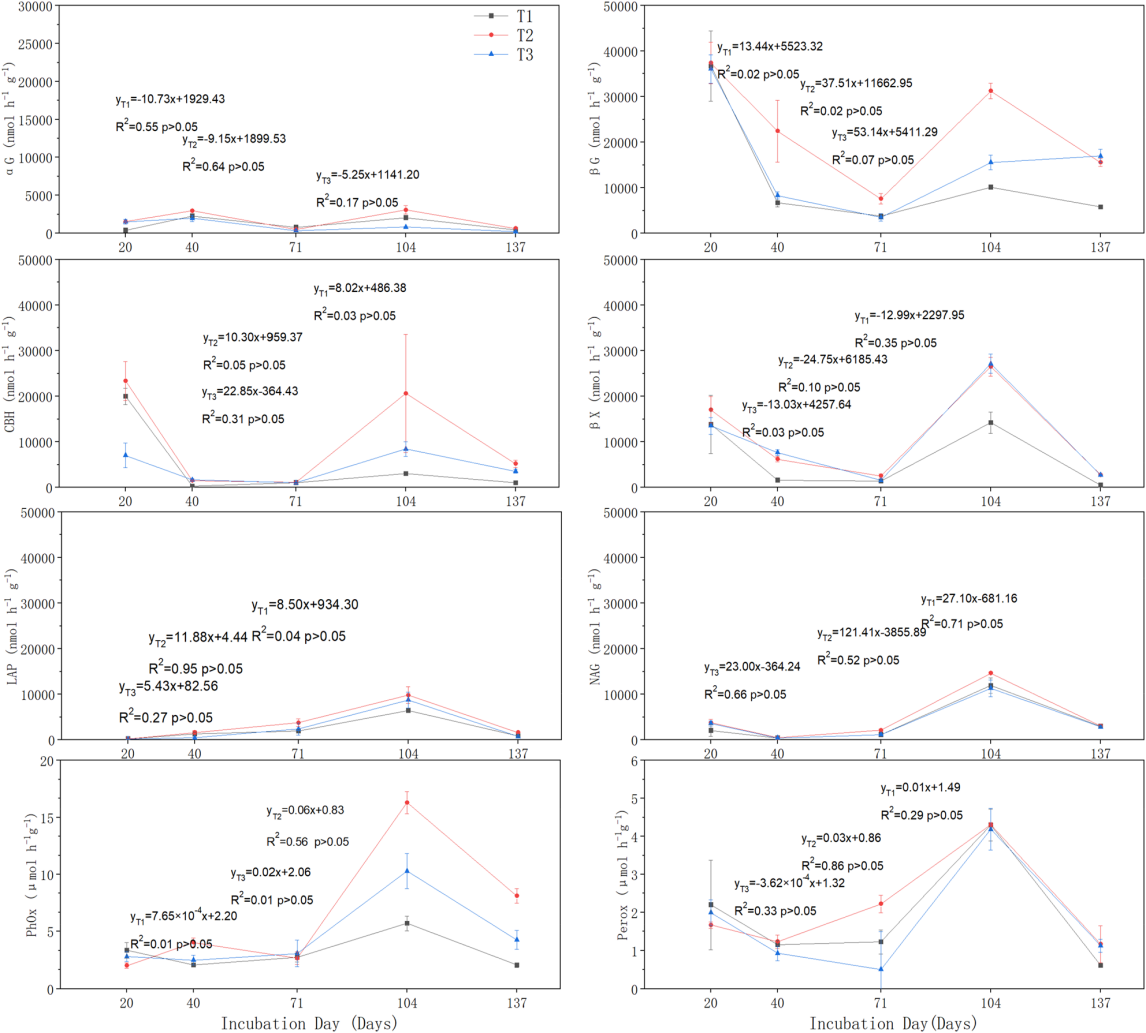
Extracellular enzyme activities of straw returned to the field in different returning periods.

The activity of extracellular enzymes for straw decay in the returned straw produced significant fluctuations with the time of return, with all eight extracellular enzymes showing high activity at 104 d of return and a significant decrease at the later stage of return (137 d) (Fig. 5). Among them, the extracellular enzymes related to the C cycle (βG, CBH, βX) showed a more consistent trend throughout the experimental period, i.e., the extracellular enzyme activities were higher at the beginning of straw return (20 d), and then (20–71 d of return), the extracellular enzyme activities gradually decreased, and then increased significantly after 71 d of straw return. Extracellular enzyme activities related to the N cycle (LAP, NAG) and redox reactions (PhOx, Perox) were low in the middle and early stages of straw return (before 71 d of return) and then increased significantly in the next 30 d. The activity of extracellular enzymes related to redox reactions (PhOx, Perox) was low and the activity of extracellular enzymes related to the C cycle (βG, CBH, βX) was generally high in all treatments (Fig. 5).

**Figure 6:**
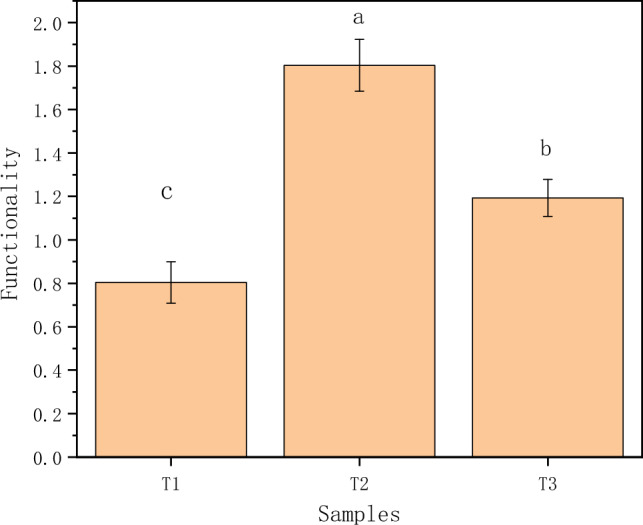
Effect of straw on potential soil functionality at different return rates.

In general, the decomposition process of returned straw was generally higher for the eight extracellular enzymes in the T2 (2/3 amount of straw returned to the field) treatment, while the T3 (full amount of straw returned to the field) and T1 (1/3 amount of straw returned to the field) treatments were higher and lower in some enzyme activity indicators. The potential functionality of the T2 treatment was significantly higher than that of the T1 and T3 treatments, while that of the T3 treatment was significantly higher than that of the T1 treatment (Fig. 6).

### Effects of straw decomposition on soil functionality by returning straw to the field

**Figure 7:**
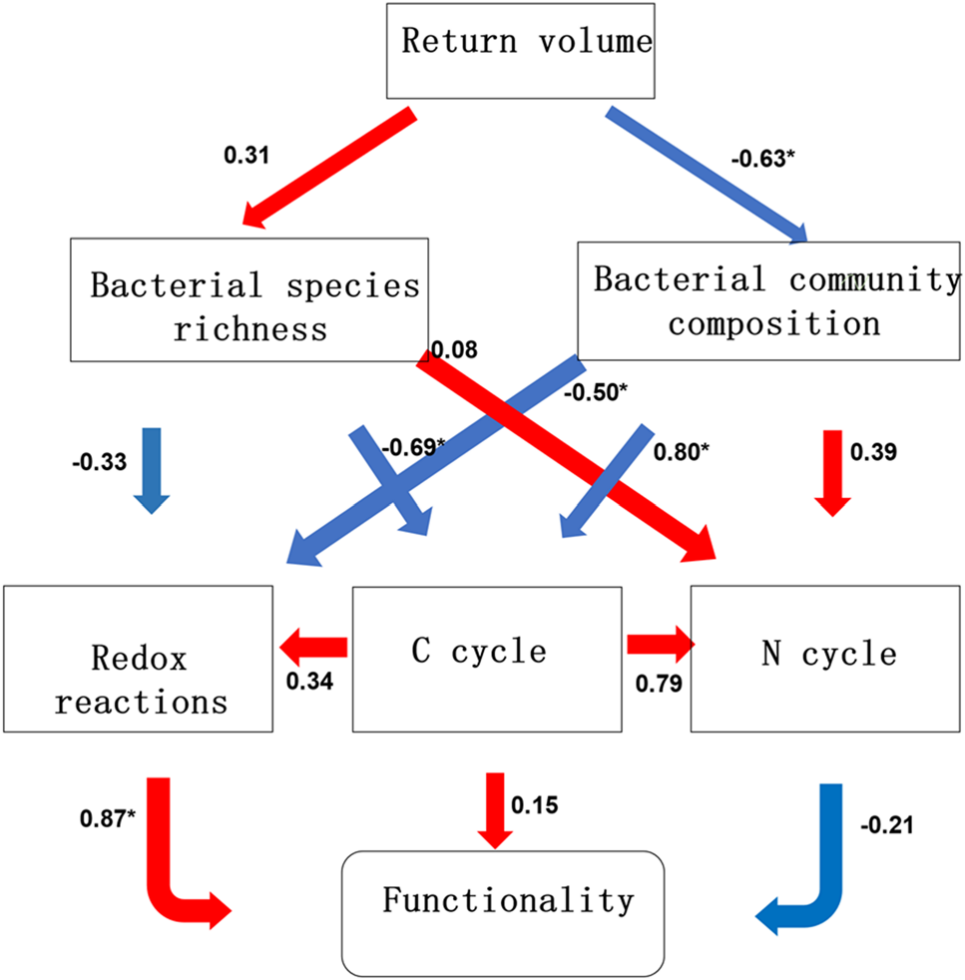
Decomposition mechanism of returned straw. The numbers adjacent to the arrows indicate coefficients. The red and blue lines indicate positive and negative relationships, respectively. The mean method was used to calculate the multifunctionality of the soil. Significance is indicated by *P < 0.05 respectively.

To better integrate the interrelationships between the amount of straw returned to the field, bacterial community, functional cycling, and due to soil multifunctionality, and to more clearly understand the effects on soil functionality due to the decomposition of returned straw, we constructed a partial least squares pathway model (PLS-PM) (Fig. 7). As shown in Fig. 7, the indirect effects of different straw volumes of returned straw on soil multifunctionality under conventional N application (130 kg hm^−2^) were caused by changes in soil bacterial species richness and bacterial composition. The amount of straw returned to the field had a more positive effect on bacterial species richness during decomposition, but had a significant negative relationship with bacterial community structure. Both bacterial species richness and community composition had positive effects on the N cycle but had negative relationships with redox reactions and the C cycle. The C cycle is an important functional cycle in the decomposition of returned straw, which can improve the potential functionality of the soil, and has a positive effect on the redox reaction and N cycle, with a coefficient of 0.79 for the N cycle. The enhancement of the redox reaction can significantly improve the potential functionality of the soil.

## Discussion

### Effect of the amount of straw returned to the field in the cool zone on the community structure of decomposing bacteria

It has been found that soil bacteria will be more sensitive to changes caused by agricultural production practices compared to fungi. Long-term biochemical and biological responses of soil bacteria to fertilization practices play a more active role in maintaining nutrient turnover and land use sustainability than fungi^31^. Returned straw decay microorganisms vary based on the material composition of returned straw at different times (Figs. 2, 4b). The relative abundance of anaerobic bacteria such as *Paludibacteraceae* was high in the early stage of decomposition (20–40 d) in this experiment, which was caused by the flooding conditions of the paddy field during this period (Figs. 1, 4b); anaerobic cordyceps that decompose cellulose, hemicellulose and other materials (Anaerolineaceae) were the dominant bacteria in the middle stage of decomposition (Fig. 4b), indicating that cellulose and hemicellulose in straw began to be decomposed in the middle stage of decomposition; in the late stage of decomposition (104–137 d), the relative abundance of *Anaerolineaceae* gradually decreased, while the relative abundance of *Clostridia 1*, which are associated with esters and alcohols gradually increased in relative abundance (Fig. 4b), indicating that the microorganisms had switched to decomposing difficult-to-use compounds such as phenolics during this period^32,33^.

Crop residues not only increase the content of soil organic carbon but also promote carbon cycling. Microorganisms are the most active group in agroecosystems, and the different microbial preferences for straw decomposition in the returned straw under different C/N conditions make the difference in their impact on the functional cycle^34,35^. Although *Ruminococcaceae*, responsible for cellulose decomposition, and *Archaeaceae*, responsible for decomposing organic matter, were the dominant bacteria common to each treatment during decomposition in this experiment, there were some differences in relative abundance, with the relative abundance of *Archaeaceae* in the T2 treatment being about three times higher than that in the T1 and The relative abundance of *Archangiaceae* in T2 treatment was about three times higher than that in T1 and T3 treatments (Fig. 5a), showing stronger potential for straw decomposition and soil function enhancement.

### Effect of straw return volume on straw extracellular enzyme activity in cool zone

Nutrient release from returned straw is an organic matter decomposition process mediated by heterotrophic microorganisms^36^. Heterotrophic microorganisms secrete hydrolases and oxidases that break down organic matter such as lignin and cellulose that limit straw decay^37^. Hydrolytic enzymes are mainly released and decomposed by binding to particulate organic matter and accompanying the decomposition of particulate organic matter with extracellular enzymes^38^. In the early stage of straw decomposition (20–71 d) in this experiment, the release of readily decomposable materials provided abundant substrates for early enzymatic reactions, in which the activities of βG, CBH, and βX involved in the C cycle and NAG involved in the N cycle were high, and the extracellular enzyme activities gradually decreased in the short term as the time of returning to the field increased (Fig. 5b, c, d, f). Oxidases are susceptible to specific microbial limitations and are mainly involved in the breakdown of difficult-to-use substances^33^. Among them are phenol oxidases and peroxidases, which are associated with the turnover of "recalcitrant" polyphenolic compounds^39^. Phenol oxidase (EC.1.10.3.2) has a low substrate specificity and is responsible for the degradation and mineralization of lignin and other polyphenol molecules, while Perox (EC1.11.1.7) is the main extracellular enzyme for lignin depolymerization^40^. The activities of PhOx and Perox in this experiment were much lower than the other six enzymes, and the activities were low and stable in the early stages of straw decomposition (20–71 d), and with the consumption of easily decomposable materials, both of the 2 oxidases showed elevated activity after 70 d of straw return and the highest activity at 104 d of return (Fig. 5g, h), and the changes of these 2 activities marked the decomposition of straw in the cool zone The changes of these two activities indicated the stage changes of straw decomposition in the cool zone.

Nutrient release from returned straw is greatly influenced by the soil environment^41^. Temperature and moisture indirectly affect the decomposition process of returned straw through the extracellular enzyme activity of decomposing microorganisms, and proper soil temperature is beneficial to increase extracellular enzyme activity^42^. The activities of four enzymes (αG, LAP, PhOx, Perox) were lower at the pre-and mid-term (20–71 d) of this experiment, and the activities of βG, CBH, βX, and NAG also showed this decreasing trend, which was also analyzed to be related to the higher water content and soil temperature inhibiting the enzyme activities during this period (Figs. 1 and 4). Around 104 d after the return of the straw to the field, the returned straw entered a period of rapid decomposition and all eight extracellular enzyme activities increased significantly, which was due to the influence of the more suitable soil environment in this period, where the substances not consumed in the previous period were rapidly decomposed (Fig. 5)^43^. It was found that the soil water content during this period was about 51.6% and the soil temperature was about 25 °C, which is similar to the suitable conditions for straw decay proposed by previous authors^44^.

The potential functionality of the T1 treatment was significantly lower than the other two treatments under different C/N conditions in this experiment (Fig. 6), which was analyzed to be related to the C and N effectiveness during straw decomposition, due to the low amount of straw returned to the field and the lack of C substrate inhibiting the activity of relevant extracellular enzymes. In addition, due to the low C/N of the microorganisms themselves, the rate is slower in decomposing organic matter with higher C/N^45–47^, and under straw return conditions, the microorganisms in the soil mainly use the returned straw as a carbon source, and the higher return volume will contribute to the lower efficiency of organic matter decomposition^48^, making the potential functionality of the T3 treatment also significantly lower than that of the T2 treatment (Fig. 6), which is also consistent with Hu Hongxiang et al.^49^. This is also consistent with the conclusion that the decomposition functionality of returned straw is negatively correlated with the amount of returned straw within a certain range, as proposed by Hu Hongxiang^49^.

### Impact of agricultural management practices on soil functionality

Information on changes in microbial biomass and soil microbial functional diversity (metabolic potential) is essential for understanding the role of microbial communities in the intensity and direction of organic matter conversion and nutrient cycling^50^. C cycle-related enzymes in this experiment had a relatively high total positive effect on soil multifunctionality (Fig. 7). In addition, we found a positive effect between C cycle-related enzymes and N and O cycle-related extracellular enzymes (Fig. 7). These findings suggest that the positive effect of microbial diversity on soil multifunctionality may be due to the activation of the C cycle. Different amounts of straw return under the same N application changed the C/N in the returned straw environment, and the input of plant residues not only increased the soil organic carbon content but also changed the soil also stimulated the metabolism of microorganisms, prompting them to further participate in the carbon cycle metabolism in the soil. Microorganisms are the most active group in terrestrial ecosystems, and returned straw-decomposing microorganisms are an important driver of the organic carbon cycle in returned straw^51^, and microorganisms are very sensitive to changes in the soil microcosm and external climate and can indicate potential functional changes in the soil at an earlier stage^35^. The straw-rotting bacteria in this experiment were very sensitive to the amount of straw returned to the field and regulated C, N, and O cycling metabolism by altering bacterial diversity and community structure, where bacterial community composition was extremely significantly influenced by the return measures (Fig. 7). However, the important role of carbon cycling in the relationship between microbial diversity and multifunctionality needs to be better explored in future studies through multi-site year field experiments.

## Conclusions

Different straw return measures can affect the metabolic process of straw decay in different ways, which in turn leads to changes in soil functionality and productivity. This study showed that the degradation of returned straw in paddy fields in the cool zone of Northeast China was mainly concentrated in the early stage (0–20 d), and the degradation-related extracellular enzyme activity was higher in the middle and late stage (104 d), and the potential soil functionality differed significantly between different straw return amounts. 2/3 of the straw return amount was the best straw return amount for the conventional N application (130 kg hm^−2^) in the cool zone. The amount of straw returned to the field directly affected the species richness and species community composition of straw-decomposing bacteria. At 1/3–2/3 of the straw return rate, the increase in the amount of returned straw was beneficial to indirectly improve the potential soil functionality.

## Data Availability

The datasets generated during and/or analyzed during the current study are available from the corresponding author on reasonable request.
